# Assessing the Utilization of Large Language Model Chatbots for Educational Purposes by Medical Teachers: A Nationwide Survey From India

**DOI:** 10.7759/cureus.73484

**Published:** 2024-11-11

**Authors:** Asitava Deb Roy, Ichchhit Bharat Jaiswal, Devendra Nath Tiu, Dipmala Das, Shaikat Mondal, Joshil Kumar Behera, Himel Mondal

**Affiliations:** 1 Pathology, Mata Gujri Memorial Medical College, Kishanganj, IND; 2 Dermatology, Mata Gujri Memorial Medical College, Kishanganj, IND; 3 Physiology, Sheikh Bhikhari Medical College, Hazaribagh, IND; 4 Microbiology, Mata Gujri Memorial Medical College, Kishanganj, IND; 5 Physiology, Raiganj Government Medical College, Raiganj, IND; 6 Physiology, Nagaland Institute of Medical Sciences and Research, Kohima, IND; 7 Physiology, All India Institute of Medical Sciences, Deoghar, Deoghar, IND

**Keywords:** ai chatbot, artificial intelligence, chatgpt, developing countries, india, large language model, medical education, medical student, medical teacher, teaching materials

## Abstract

Background

Large language models (LLMs) are increasingly explored in healthcare and education. In medical education, they hold the potential to enhance learning by supporting personalized teaching, resource development, and student engagement. However, LLM use also raises concerns about ethics, accuracy, and reliance. Understanding how educators leverage LLMs can help assess their role and implications in medical education.

Methods

This cross-sectional online survey was conducted among medical teachers in India from December 2023 to March 2024. A validated questionnaire with acceptable internal consistency and test-retest reliability was used. It collected data on LLM chatbot usage patterns, as well as teachers' knowledge, attitudes, and practices regarding LLMs for educational purposes.

Results

A total of 396 medical teachers with an average teaching experience of 4.12±2.47 (minimum six months, maximum 13 years) years participated from different parts of India. The majority of the teachers heard about ChatGPT (OpenAI, San Francisco, CA, USA) (85%), followed by Copilot/Bing (Microsoft, Washington, DC, USA) (53%), and Gemini/Bard (Google, Mountain View, CA, USA) (45%) (p-value < 0.0001). However, 29% of the respondents never used it and 47% rarely use LLMs for educational purposes (p-value < 0.0001). The majority of the teachers use it for making any topic simple (55%), generating text for PowerPoint slides (55%), generating multiple-choice questions (MCQs) (52%), and finding answers to student’s queries (35%). Knowledge (3.4±0.47) showed the highest score, followed by practice (3.3±0.81) and attitude (3.14±0.46) (p-value = 0.0023).

Conclusion

While awareness of LLMs was high among medical teachers in India, their actual usage for educational purposes remains limited. Despite recognizing the potential of LLMs for simplifying topics, generating teaching materials, and addressing student queries, a significant proportion of educators seldom integrate these technologies into their teaching practices. Institutions may provide training to help medical educators effectively integrate LLMs into teaching practices.

## Introduction

The integration of technology into medical education has recently accelerated, especially with the rise of artificial intelligence (AI) applications [1]. While predictive AI has long been a part of education and healthcare, the introduction of generative AI is now transforming these fields on a global scale [2]. Large language model (LLM) chatbots have been tested across various educational and healthcare contexts [3]. These chatbots have shown capabilities including answering complex medical essay-type questions, handling multiple-choice questions (MCQs), interpreting clinical vignettes, assisting physicians in generating educational content, simplifying complex medical topics, and drafting or revising medical reports [4-9].

Medical education is an ever-evolving field, continually seeking innovative approaches to improve the learning experience [10]. However, medical teachers in developing countries, like India, face significant challenges. Limited resources, such as outdated infrastructure and insufficient funding, can hinder the delivery of quality education. Additionally, a shortage of qualified faculty and high student-to-teacher ratios can strain teaching quality and reduce opportunities for personalized attention [11,12]. Rapid advancements in medical knowledge and technology demand ongoing professional development, yet access to training and resources may be limited. Moreover, bureaucratic challenges and administrative tasks often divert valuable time and focus away from teaching and research efforts [13].

LLM chatbots, characterized by their ability to understand and generate human-like text, offer a unique avenue for educators to engage with learners dynamically and interactively. LLMs can offer significant support to medical teachers in developing countries by providing access to up-to-date medical knowledge, educational resources, and teaching materials [14]. LLMs can aid in curriculum development, lesson planning, and creating interactive educational content tailored to the specific needs and challenges of medical education in these countries [15]. Additionally, LLMs can assist in overcoming language barriers by offering translation services and enabling medical teachers to access educational materials in various languages. By leveraging LLMs, medical teachers in developing countries can enhance the quality of education and ultimately improve healthcare outcomes for their communities [16]. A study by Hamilton in the USA found that more than 50% of the teachers who participated in the survey believe that AI has positively impacted the teaching and learning process and 60% of educators are using AI in their classrooms, while 35% use it for student support [17]. However, the extent to which medical teachers have incorporated these models into their teaching methodologies in developing countries like India remains an open question.

In this context, this study seeks to fill this gap by examining the current landscape of LLMs utilization in medical education by medical teachers. The findings of this study will help inform stakeholders about how Indian medical teachers are using LLMs and their opinions about their use in medical educational purposes. This will also provide a platform for strategies for the future integration and training of medical teachers for optimum usage of LLMs in medical educational purposes.

## Materials and methods

Study design and setting

This research employs a mixed-methods approach, integrating both quantitative and qualitative methods to thoroughly evaluate how medical teachers use LLM chatbots in medical education. Data collection was conducted online, using a survey with closed-ended questions and an open-ended question for participants to share their opinions. The study took place from October 2023 to March 2024 and received approval from the Institutional Ethics Committee of Mata Gujri Memorial Medical College, Kishanganj, Bihar, India (MGM/IEC-08/2024).

Sample size

Assuming a target population of 276,415 [18] a confidence level of 95% (corresponding to a Z-score of 1.96), an estimated proportion of 0.5 for maximum variability, and a margin of error of 5%, the sample size was 384.16. Rounding up to the nearest whole number, the recommended sample size was approximately 385.

Participants

The target participants were any medical teacher from any institution (government-run, private-run, and trustee-managed) and specialties. Participants were recruited through purposive (convenience) and snowball sampling to recruit a maximum number of participants.

Survey tool

For this study, we used a questionnaire that contained a total of three parts. The first part of the questionnaire collected information about the sex, teaching experience (after post-graduation) in years, state of the teaching institution, the name of LLM they heard of what LLM they use, what purpose they use, and how often they use it. The second part contained seven statements for knowledge (i.e., awareness), six for attitude, and eight for practice. The third part contained an open question to inform about anything they would like to share in this context. We designed this questionnaire after reviewing the currently available literature [19-22]. The content validity was checked by three experts and the questionnaire was pre-tested on a sample of 30 participants. Respondents were interviewed about their understanding of the questionnaire. From their input, we added more examples of LLM chatbots in the first question of the knowledge domain and added examples of search engines (Google) in the fourth question of the practice domain for better understanding. The responses were coded and used for the calculation of Cronbach’s alpha. The internal consistency for the knowledge domain was 0.74, attitude was 0.86, and practice was 0.79. The same participants were asked to respond after two weeks, and the scores were used to calculate the test-retest reliability. The calculated intraclass correlation coefficient (ICC) for the knowledge domain was 0.78, attitude was 0.67, and practice was 0.73. As the questionnaire has acceptable internal consistency and reliability, we used the questionnaire. The questionnaire is available in Appendices (Tables 2, 3).

Data collection

The questionnaire was made available on Google Forms platforms. The questionnaire has an informed consent part that has a compulsory question to answer with only one response. Without agreeing on the response (I agree to participate voluntarily), the form would not go forward to the survey proper. All the questions were compulsory. Hence, we received all completed responses from the participants. The survey links were shared in various social media groups (WhatsApp, Facebook, and Telegram (Meta, USA)) known to the authors (convenience). The social media message with the survey link had a request to share the survey links with medical teachers (snowballing). The survey started in December 2023 and ended in March 2024. We have sent three reminders and requests for participation in the survey during this period to reach the desired sample size.

Data analysis

Collected data were presented in numbers, percentages, and mean and standard deviations. Any categorical data were compared by Chi-square or Fisher’s exact test according to data distribution. Survey data were also coded (strongly agree = 5, agree = 4, neutral = 3, disagree = 2, strongly disagree = 1) to compare the scores among the three domains of knowledge, attitude, and practice. We used GraphPad Prism 9.5.0 (GraphPad Software Inc., USA) for statistical analysis. The textual data received as an additional comment was analyzed thematically to identify further insight provided by the respondents. The textual data was analyzed by QDA Miner Lite (Provalis Research, Montreal, Canada) and a word cloud was generated to show the most occurring words in the text we received as comments from the teachers.

## Results

**Figure 1:**
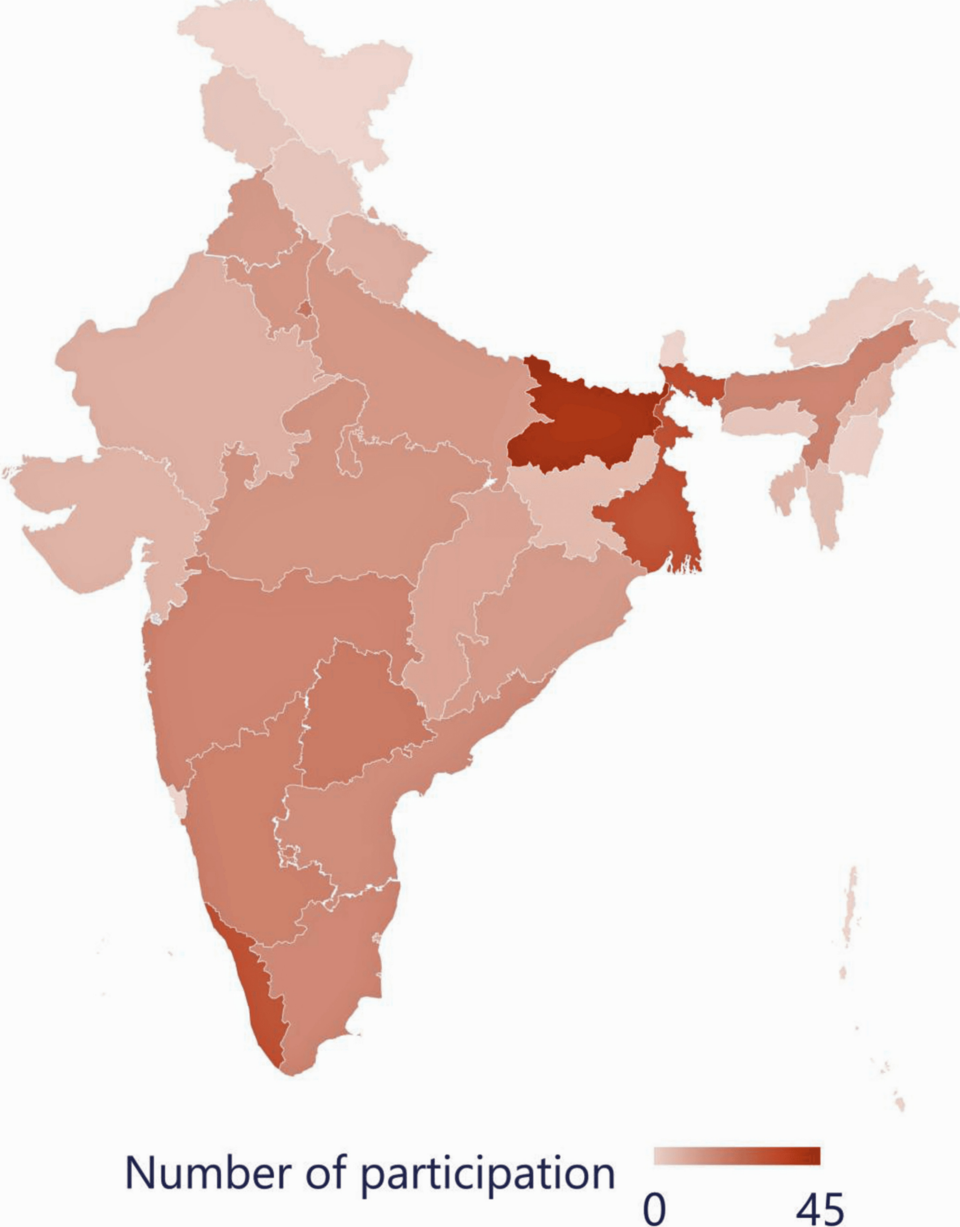
Distribution of survey participants by Indian states (n = 396) This map was created by Dr. Himel Mondal on Microsoft Excel 2021

A total of 396 medical teachers participated in the study, comprising 212 males (53.54%) and 184 females (46.46%), with an average teaching experience of 4.12±2.47 years (ranging from six months to 13 years). Of the participants, 362 (91.41%) held a Bachelor of Medicine and Bachelor of Surgery (MBBS) and a Doctor of Medicine (MD) postgraduate degree, 20 (5.05%) held an MBBS and a Diplomate of National Board (DNB) postgraduate degree, 11 (2.78%) had a Master of Science (MSc), and three (0.76%) had a Doctor of Philosophy (PhD) in medical sciences. The top three states with the highest number of participants were Bihar (45, 11.36%), West Bengal (33, 8.33%), and Kerala (32, 8.08%). Figure 1 illustrates the relative number of participants from each state.

**Figure 2:**
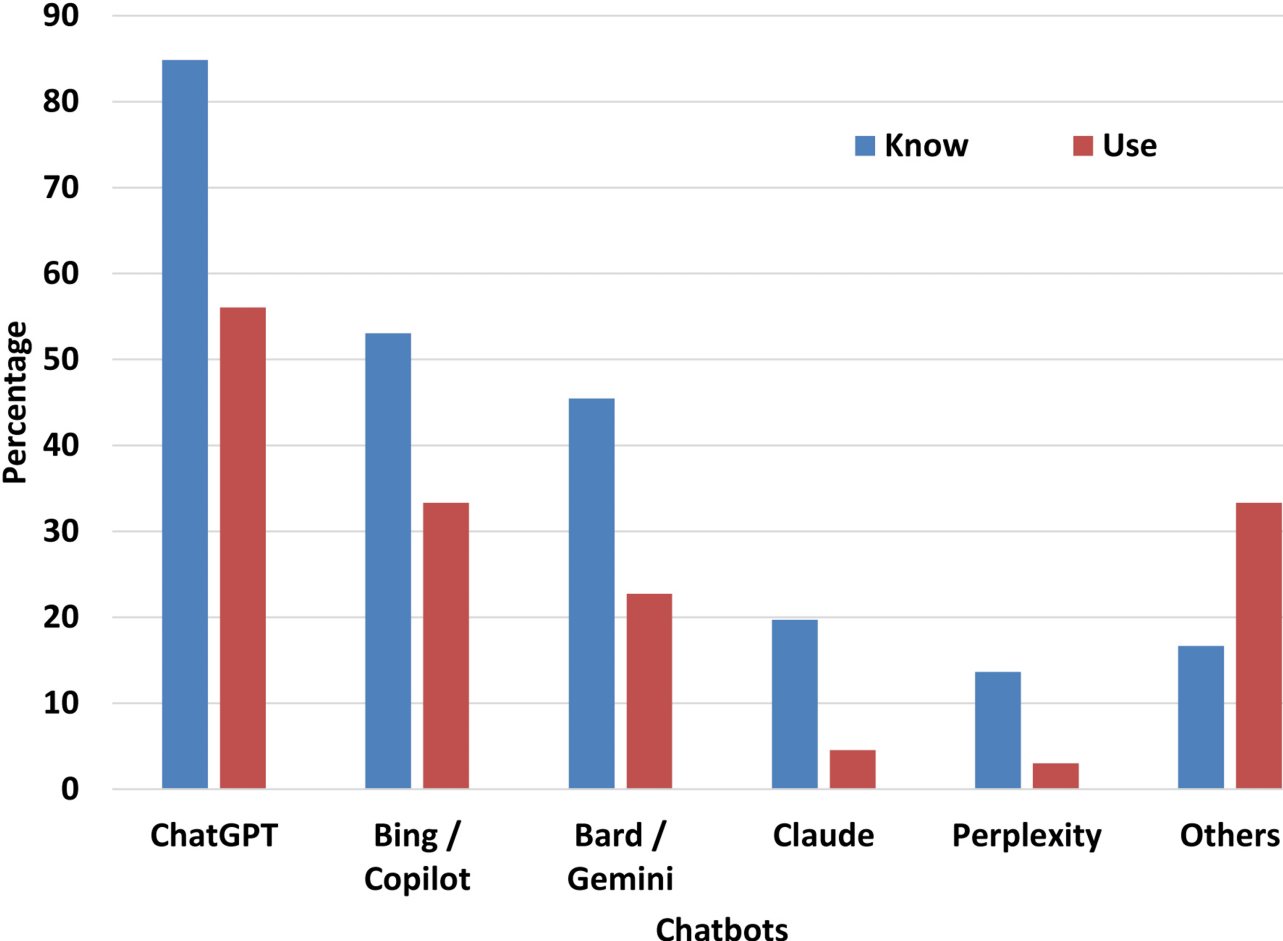
Percentage of respondents according to knowledge and use The respondents (n = 396) had an option to choose multiple large language model chatbot Statistical test: Chi-square (5) = 97.9, p-value < 0.0001

Among the various available chatbots, most teachers reported being familiar with ChatGPT (OpenAI, San Francisco, CA, USA), followed by Copilot (Microsoft, Washington, DC, USA) and Gemini (Google, Mountain View, CA, USA). Figure 2 shows the distribution of teachers' awareness and use of LLM chatbots for educational purposes.

**Figure 3:**
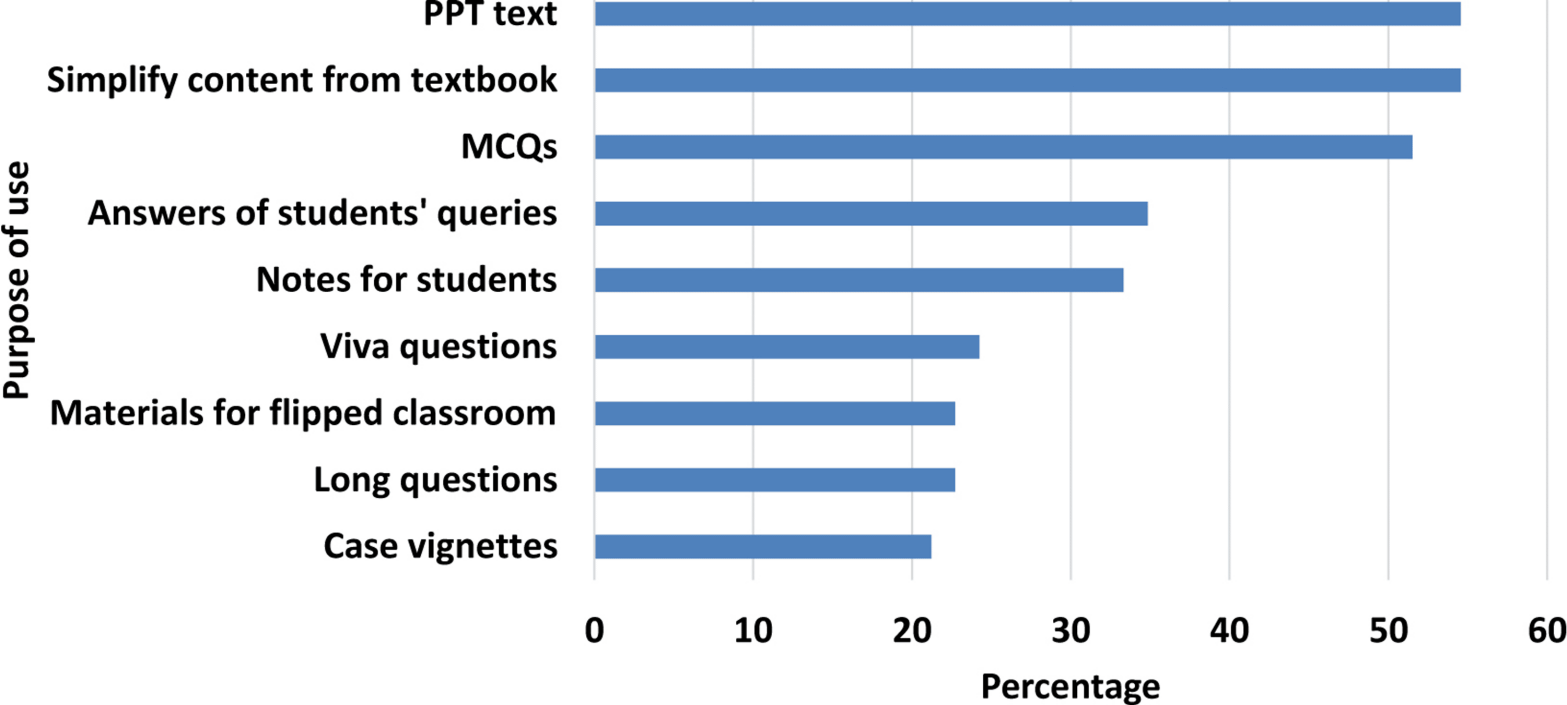
Purpose of large language model usage among medical teachers as a percentage of total respondents PPT: PowerPoint presentation, MCQ: Multiple-choice question Statistical test: Chi-square (5) = 77.47, p-value <0.0001

The majority of teachers reported using LLM chatbots for simplifying topics (218, 55.05%), creating PowerPoint slides (218, 55.05%), generating MCQs (206, 52.02%), and answering students' queries (139, 35.1%). Figure 3 illustrates the various purposes for which teachers use LLM chatbots.

**Figure 4:**
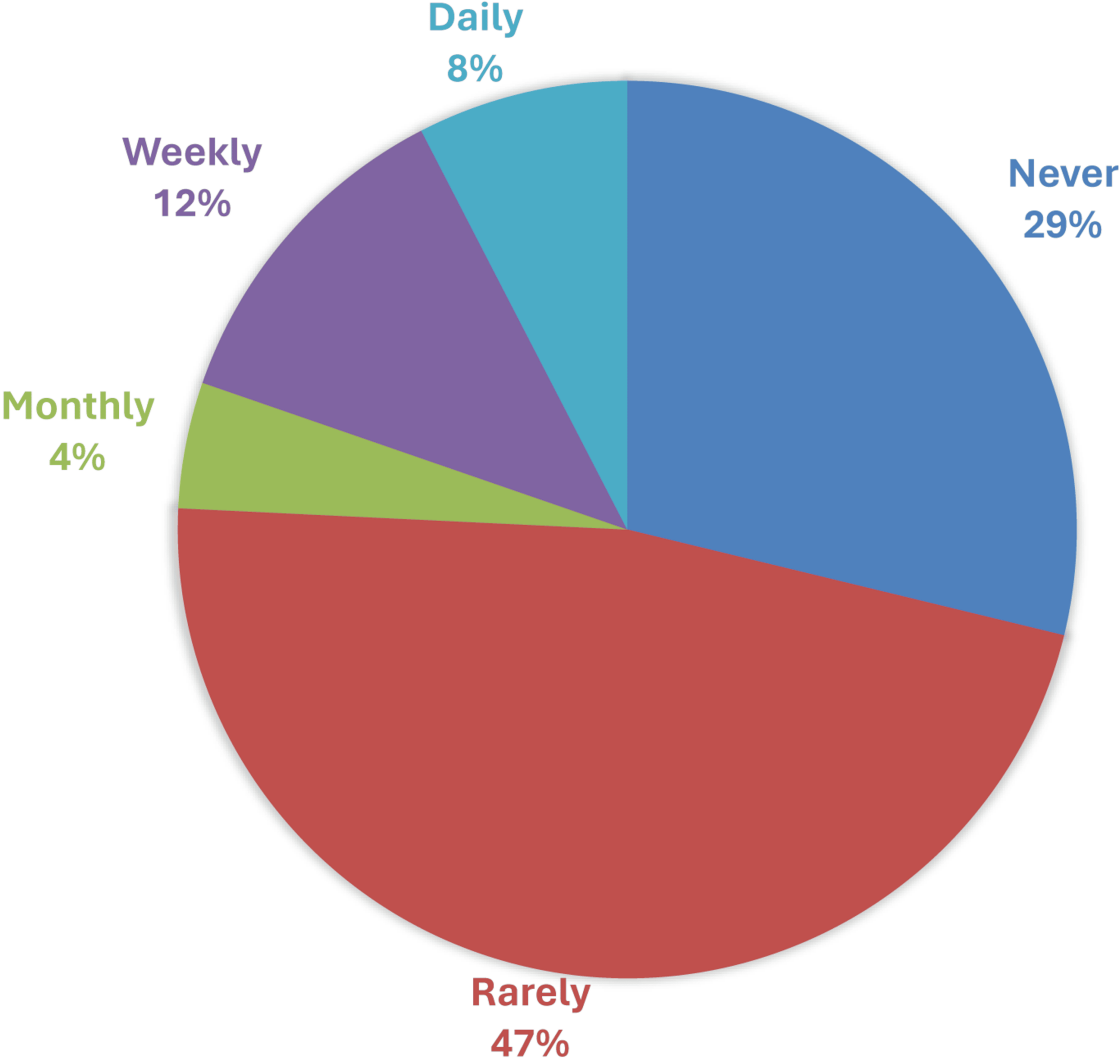
Frequency of large language model chatbot use among medical teachers for educational purposes Statistical test: Chi-square (4) = 249.5, p-value < 0.0001

An analysis of usage frequency revealed that 115 respondents (29.04%) never use LLM chatbots, while 186 (46.97%) reported rarely using them for educational purposes. The distribution of usage frequency is shown in Figure 4.

**Figure 5:**
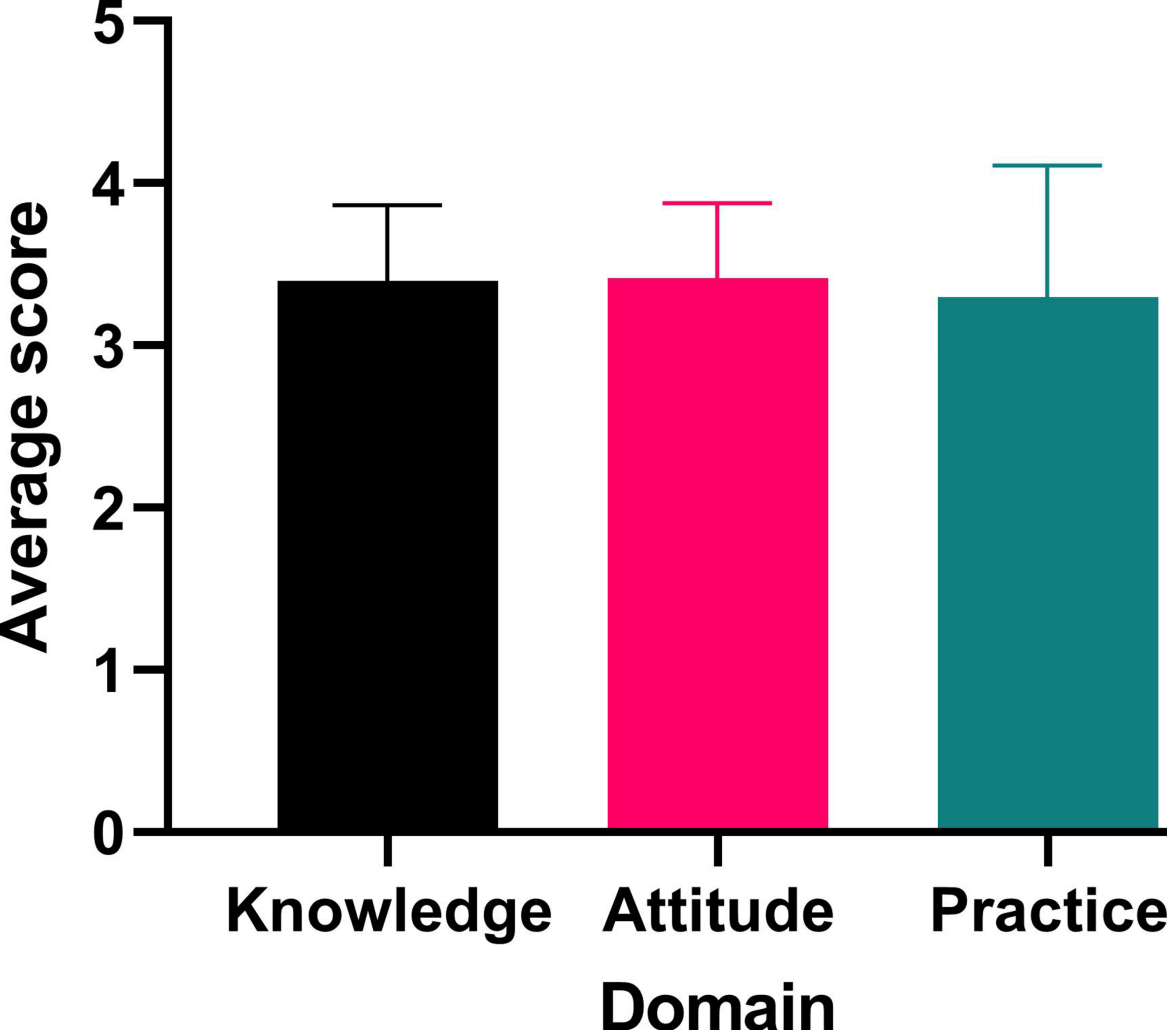
Average score of knowledge, attitude, and practice Statistical test: ANOVA F = 6.72, p-value = 0.0023

When the responses were coded and compared, the highest score was observed in the knowledge domain (3.4±0.47), followed by practice (3.3±0.81), and attitude (3.14±0.46). Post-hoc tests revealed significant differences between two pairs: knowledge vs. practice (p-value = 0.037) and attitude vs. practice (p-value = 0.002). The average scores are shown in Figure 5.

In a linear regression model, the practice of using LLMs was predicted by overall levels of knowledge and attitude (*r* = 0.582, R² = 0.339, F = 16.123, p-value < 0.0001). This suggests that higher familiarity with and positive attitudes toward LLMs correlate with more frequent or effective use of these tools, highlighting both the importance of fostering understanding and favorable perceptions of LLMs in educational settings. However, when analyzed individually, the contribution of knowledge was not significant (t = 1.719, p-value = 0.09), while the contribution of attitude was significant (t = 4.52, p-value < 0.0001) in predicting the practice of LLMs.

A total of 23 participants responded to the open-ended questions. Two authors independently extracted themes using QDA Miner Lite, and a consensus was reached to finalize these themes. Our analysis revealed six prominent themes, which are presented in Table 1.

**Table 1:** Themes and their brief with direct quotes from the opne-ended opinion of the survey participants (n = 23) LLM: Large language model, NMC: National medical Council (a central body regulating medical education in India)

Theme	Description	Quoted text
Training	Emphasis on the need for training, especially for those who are not tech-savvy. Desire for guidance on learning LLM usage.	"I must learn first, then to apply. Where should I learn?"
Limitations	Concerns about incorrect or biased information, reliance on proprietary models, downtime, and data privacy issues.	"The tools are not ready in any stretch at this moment"
Supplementary role	LLMs seen as tools to complement traditional resources, not replace them. Helpful in simplifying concepts and providing resources.	"Large language models are an adjuvant but not the entirety of medical education, it should be treated and used likewise"
Practical utility	Useful for quick information retrieval.	"I use it to get information precisely when Google can't help”
Ethical concerns	Worries about ethical issues related to data sourcing and scraping, suggesting locally run models for privacy and reliability.	"A locally run finetuned model can be better suited for medical purposes where it can run more reliably and securely."
Adoption	Call for official endorsement and structured implementation by governing bodies like the NMC.	"LLM should be used routinely for medical teaching and training, only when permitted in writing by the NMC"

Participants expressed a need for “training” to enhance their understanding of using LLMs for medical education. Conversely, some participants highlighted the importance of considering the “limitations” of LLMs before incorporating them into educational settings, emphasizing the potential shortcomings and constraints associated with their use. Many viewed these models as “supplementary” tools that can aid traditional teaching methodologies. Several teachers mentioned that they typically use LLMs only when traditional internet searches do not yield the desired content. Additionally, many medical teachers voiced concerns about the information they share with chatbots, citing “ethical and practical concerns” regarding the sharing of sensitive medical data. As a result, they expressed a desire to obtain approval from regulatory bodies before adopting LLMs for educational purposes.

## Discussion

We found that most medical teachers are familiar with various LLM chatbots like ChatGPT, Copilot, and Gemini. There was a predictive relationship suggesting that overall, the knowledge and attitude toward LLMs could lead to their use in education. This highlights the importance of enhancing teachers' understanding of LLMs to encourage their practical application in education. As technology is evolving, advances in digital education will help equip doctors in practice [23].

Our study indicates that medical teachers predominantly use LLMs for generating PowerPoint text and simplifying textbook content. Additionally, these tools are frequently employed for creating MCQs, responding to students' queries, and preparing notes. Other educational applications include generating viva and long questions, materials for flipped classrooms, and case vignettes, albeit to a lesser extent. Although the percentage of teachers using AI for educational purposes is less than that of the USA, these findings highlight the versatility of LLMs in supporting various aspects of medical education and provide a base for future adoption in developing countries [24].

A significant portion of medical teachers either never or rarely use LLMs for educational purposes. A smaller fraction utilizes these tools on a weekly or daily basis. This pattern suggests that while awareness of LLMs is relatively widespread, their integration into regular teaching practices is still limited. Several factors may contribute to this finding. Infrastructure challenges, including inadequate internet connectivity and access to technology, may hinder widespread adoption. A lack of familiarity or training with AI platforms could deter teachers from incorporating them into their educational practices. Potential solutions include investing in high-speed internet and updated equipment and securing targeted funding for digital infrastructure. We also found in this study that teachers would like to get training for it. Hence, it can be provided by institutions. Cultural factors, such as traditional teaching methods, may also play a role. Moreover, concerns about data privacy and security may lead to hesitancy in utilizing digital platforms for educational purposes. Supporting our finding, the study by Salih et al. at Jazan University's Faculty of Medicine reveals generally positive opinions about integrating AI into medical education. However, faculty commented that ethical concerns like academic integrity, information accuracy, plagiarism, intellectual property, privacy, and cultural sensitivity need addressing before AI adoption [25].

In the knowledge domain, medical teachers show a broad understanding of LLMs like ChatGPT, Google Bard (now known as Gemini), Microsoft Bing (now known as Copilot), and Perplexity, recognizing them as examples of generative AI. While many educators acknowledge that LLMs can generate both incorrect and biased content, there is also an awareness of their potential to simplify complex medical concepts and complement traditional educational materials. However, understanding the mechanics of how these models generate information varies, with some educators confident in their understanding and others less certain. Regarding attitudes toward LLMs, there is a general openness to incorporating these tools as supplementary teaching aids. Many teachers believe that LLMs could positively transform medical education and support their use by medical colleges. However, there are concerns about over-reliance on these models potentially hindering the development of traditional teaching skills and the risk of disseminating incorrect information. In terms of practical application, while some educators regularly use LLMs to obtain clearer explanations and discover new resources, others only turn to these tools when traditional sources or search engines fall short. The integration of LLMs into class material preparation is still relatively limited, indicating room for growth in practical adoption [26,27].

From the qualitative data analysis, we found that the teachers perceive the need for formal training in using LLMs for educational purposes. Many teachers are unfamiliar with LLM functionalities, creating a technological gap between their current skill set and the rapid development of these tools. Training would bridge this gap and build confidence in integrating LLMs into teaching methods. Along with teachers, students also seek the need for training as reported by Civaner et al. from Turkey [28]. Knowledge and attitude significantly influence the use of LLMs in medical education. Teachers with more knowledge and positive attitudes toward technology are more likely to embrace and utilize LLMs effectively. Addressing misconceptions and providing accurate information can reduce barriers to adoption. Data privacy and ethical concerns about proprietary models are prominent among educators. Teachers worry about the handling of sensitive student and patient data, particularly the ethical implications of data scraping without explicit consent. The lack of transparency in how data is used and stored by these models exacerbates mistrust. Gordon et al. emphasized the need for establishing ethical principles in the implementation of AI in medical education [29]. Due to privacy concerns, teachers prefer locally hosted models. Locally hosted models offer greater control over data, ensuring it is not exposed to external entities and can align better with institutional and regulatory compliance requirements. Buabbas et al. advised the curriculum developers to include AI instruction or training in undergraduate medical programs [30]. Teachers in our study also call for structured adoption and policy regulation. Standardized guidelines and endorsement by authoritative bodies like the National Medical Commission (NMC) can provide legitimacy and trust in using LLMs. A clear policy framework can address ethical, legal, and operational concerns, facilitating smoother integration of LLMs into educational practices.

The findings of the study may be considered by various stakeholders involved in the medical education sector. This is the first nationwide study to explore the teachers’ knowledge, attitude, and practice of using LLM for medical education. By understanding the benefits and challenges associated with these advanced technological tools, educators can enhance their pedagogical strategies, creating a more engaging and dynamic learning environment for medical students. The study underscores the need for comprehensive training programs to enhance teachers' familiarity and proficiency with LLMs, addressing the current technological gap [1].

Several limitations of this study should be considered. This was a cross-sectional study and reflects only the picture of a limited time. The LLMs and the mindset of teachers are changing. The sample was a convenience sample and may not represent all the teachers as we only surveyed a limited number of teachers. In addition, the respondents were majorly early career educators. In addition, this was an online survey shared via social media messengers. Hence, we had limited information about the credibility of the participants. In addition, to make the survey anonymous, we did not collect any identifying of respondents. Further studies with multicentric surveys with random institution selection from Indian medical institutions would provide more generalizable results.

## Conclusions

This nationwide survey of medical teachers in India reveals a cautious yet hopeful outlook toward the integration of LLMs in medical education. While many educators acknowledge the potential of LLMs to enhance teaching by simplifying complex concepts and providing supplementary resources, there are significant concerns about their reliability, accuracy, and ethical implications. The study highlights the urgent need for structured training programs to improve familiarity and effective usage of LLMs among medical teachers. Additionally, there is a strong call for regulatory oversight by authoritative bodies such as the NMC to ensure the safe and ethical implementation of these technologies. While LLMs are not yet seen as a replacement for traditional educational tools, they hold promise as valuable adjuncts in the evolving landscape of medical education.

## Appendices

Survey questionnaire

This is an anonymous survey, and we only collect your sex, teaching experience, and the state in which your college is situated. We are not collecting your email address or any other identity information. Participation in this study is completely voluntary. If you decide not to participate, you can leave the form here and exit.  Please be aware that if you choose to participate, and later do not want to submit the response, you may stop participating at any time. We shall try our best to maintain the confidentiality of the research records or data, and all data will be destroyed after complete analysis. By clicking on the “Agree” button, you are indicating that you have read the above text and participating in this survey voluntarily and without any coercion.

The form contains a total of seven questions in part 1 (Table 2) and 21 statements in part 2 (Table 3) with an open question for additional comments. Each statement has five response options ranging from “strongly agree” to “strongly disagree.” Please select a response option against each statement. If you have any questions or would like a copy of this consent letter, please contact me at (email address). Thank you in advance for your participation!

**Table 2:** Basic information of the respondent and use of large language model chatbots LLM: Large language model, MCQ: Multiple choice question

Chacteristics/question	Options
Sex	Male/Female/Other
Teaching experience after post-graduation	….. (years)
State where you teach	….. (Indian state; e.g., West Bengal)
Name of LLM chatbot you heard of	….. (you can name multiple)
What LLM chatbot do you use?	….. (you can name multiple)
How often do you use it?	Never/Rarely/Monthly/Weekly/Daily
For what purposes do you use it?	Preparing PPT text/Preparing notes for students/Preparing simplified content from complex topics/Preparing materials for Flipped classroom/Preparing case vignettes/Preparing long questions/Preparing MCQs/Finding answers to students' queries/Preparing viva questions

**Table 3:** Questionnaire to assess the knowledge, attitude, and practice with a open question GPT: Generative pre-trained transformer, e-books: electronic books

Domain	Statement	Response options				
		Strongly agree	Agree	Neutral	Disagree	Strongly disagree
Knowledge	ChatGPT, Google Bard/Gemini, Microsoft Bing/Copilot, or Perplexity are examples of generative artificial intelligence					
	Large language models are one type of generative artificial intelligence					
	I understand how large language models generate information and responses					
	Large language models can generate wrong information					
	Large language models can generate biased content					
	Using large language models can help simplify complicated medical concepts					
	Large language models can help teachers along with traditional learning materials like textbooks, notes, e-books, etc.					
Attitude	I am open to including large language models as extra teaching tools for medical students					
	Large language models provide medical information that may be helpful for teaching					
	Medical Colleges should promote the use of large language models in the teaching-learning process					
	Large language models could change how I teach and access medical knowledge					
	Relying on large language models will not affect my teaching skills					
	I do not trust generated content and will check before using it for teaching purposes					
Practice	I often use large language models to get clearer explanations of medical topics I'm preparing to teach					
	Large language models have shown me new resources and references for my teaching					
	I use large language models only when I cannot get the information in books					
	I use large language models only when I cannot get the information in Google or another search engine					
	I modify my teaching methods based on insights I get from large language models					
	Using large language models has made me more confident in talking about medical subjects					
	I share reliable medical information I get from large language models with my students					
	I use it for preparing my class materials					
Open opinion	If anything else you want to share, please write in the text box.					
